# Enhanced Hepatic apoA-I Secretion and Peripheral Efflux of Cholesterol and Phospholipid in CD36 Null Mice

**DOI:** 10.1371/journal.pone.0009906

**Published:** 2010-03-26

**Authors:** Pin Yue, Zhouji Chen, Fatiha Nassir, Carlos Bernal-Mizrachi, Brian Finck, Salman Azhar, Nada A. Abumrad

**Affiliations:** 1 Center for Human Nutrition, Department of Medicine, Washington University School of Medicine, St. Louis, Missouri, United States of America; 2 Division of Endocrinology, Department of Medicine, Washington University School of Medicine, St. Louis, Missouri, United States of America; 3 Department of Veterans Affairs, Palo Alto Health Care System, Geriatric Research, Education and Clinical Center, Palo Alto, California, United States of America; 4 Stanford University School of Medicine, Stanford, California, United States of America; University of Tor Vergata, Italy

## Abstract

CD36 facilitates oxidized low density lipoprotein uptake and is implicated in development of atherosclerotic lesions. CD36 also binds unmodified high and very low density lipoproteins (HDL, VLDL) but its role in the metabolism of these particles is unclear. Several polymorphisms in the CD36 gene were recently shown to associate with serum HDL cholesterol. To gain insight into potential mechanisms for these associations we examined HDL metabolism in CD36 null (CD36^−/−^) mice. Feeding CD36^−/−^ mice a high cholesterol diet significantly increased serum HDL, cholesterol and phospholipids, as compared to wild type mice. HDL apolipoproteins apoA-I and apoA-IV were increased and shifted to higher density HDL fractions suggesting altered particle maturation. Clearance of dual-labeled HDL was unchanged in CD36^−/−^ mice and cholesterol uptake from HDL or LDL by isolated CD36^−/−^ hepatocytes was unaltered. However, CD36^−/−^ hepatocytes had higher cholesterol and phospholipid efflux rates. In addition, expression and secretion of apoA-I and apoA-IV were increased reflecting enhanced PXR. Similar to hepatocytes, cholesterol and phospholipid efflux were enhanced in CD36^−/−^ macrophages without changes in protein levels of ABCA1, ABCG1 or SR-B1. However, biotinylation assays showed increased surface ABCA1 localization in CD36^−/−^ cells. In conclusion, CD36 influences reverse cholesterol transport and hepatic ApoA-I production. Both pathways are enhanced in CD36 deficiency, increasing HDL concentrations, which suggests the potential benefit of CD36 inhibition.

## Introduction

Cluster of Differentiation 36 (CD36) is an 88 kD transmembrane glycoprotein and type B scavenger receptor with a range of lipid ligands that includes long chain fatty acids (FA)[1] and native or modified lipoproteins[2]. As a result, CD36 is implicated in various cellular activities linked to a number of pathologies including atherosclerosis[3]. Cholesterol uptake by macrophages from oxidized low density lipoproteins (ox-LDL) or LDL promotes formation of lipid-laden “foam” cells, a critical step in atherosclerotic lesion formation. CD36 facilitates ox-LDL uptake by binding the modified lipids and internalizing the particles [4]. CD36 was also shown to delay degradation of native LDL *in vivo* [5]. These two functions would be predicted to have a pro-atherosclerotic effect.

High density lipoproteins (HDL) have an antiatherogenic effect reflecting their function in reverse cholesterol transport to the liver and their anti-oxidant properties[6]. Like SR-B1, a family member and HDL receptor[7], CD36 binds these particles with high affinity[8], [9]. However, its efficiency in mediating selective cholesteryl ester (CE) uptake is low compared to SR-B1[9], [10], which led to the concept that it has a minor if any role in HDL metabolism. In CD36 null (CD36^−/−^) mice fed a chow diet, plasma cholesterol and HDL are modestly elevated (about 30%)[11], but the significance of this observation is unclear. HDL level is influenced by that of very low density lipoproteins (VLDL) and an inverse relationship is often observed between serum levels of both lipoproteins[12]. High VLDL as a result of increased hepatic secretion[12] or diminished clearance due to lipoprotein lipase deficiency[13] usually associate with low HDL. A major phenotypic characteristic of CD36^−/−^ mice is a defect in tissue FA uptake that feedbacks to inhibit VLDL clearance[14], [15]. However, the expected inverse relationship between VLDL and HDL does not hold in the CD36^−/−^ mouse where both lipoproteins were reported to be increased[11]. Recent evidence suggested the possibility that CD36 may play a direct role in HDL metabolism. A region of chromosome 7 (7q11.2–7q21.11) encompassing the CD36 gene was linked to components of the metabolic syndrome including HDL in genome-wide linkage studies[16]. Our study in a large population sample identified strong associations between single nucleotide polymorphisms (SNPs) in the CD36 gene and serum HDL cholesterol[17]. Of thirty-six CD36 tag SNPs that were examined, nine associated with increased serum HDL-C and seven with reduced HDL-C. These associations remained significant after adjusting for triglyceride (TG) levels consistent with a direct effect. These observations suggested that the role of CD36 in HDL metabolism may be underappreciated.

In this report we describe the impact of CD36 deficiency in mice on HDL metabolism and examine the mechanisms involved. Our results implicate CD36 in reverse cholesterol transport and in hepatic secretion of HDL apolipoproteins, two processes that influence HDL levels.

## Materials and Methods

### Animals and diets

CD36^−/−^ mice[11] on the C57BL/6J genetic background were housed in a barrier facility with free access to food and water. For the high cholesterol feeding studies, 8 week-old mice were maintained on a high fat, high cholesterol (HFHC) diet (Teklad Harlan, TD.02028, 42% calories from fat, 1.25% cholesterol) for 4 weeks. For studies with isolated hepatocytes and macrophages and *in vivo* HDL clearance 12 week-old mice fed CHOW were used. All experimental protocols were performed in compliance with the standards established by the US Animal Welfare Acts, and were approved by the Animal Study Committee of Washington University School of Medicine.

### Plasma lipid measurements

Plasma lipids were measured using 12 hours fasted mice. Blood samples, 50 µl, were collected from the mouse tail vein in heparin coated capillaries, and plasma was obtained by centrifugation at 12,000 g for 5 minutes at 4°C. Plasma triglyceride (TG), total cholesterol (CHOL), HDL-cholesterol, phospholipids (PL), and free fatty acids (FA) were measured using enzymatic kits (Wako Chemicals, Richmond, VA).

Lipoprotein fractionation was conducted by fast protein liquid chromatography (FPLC) using a Superose 6 column HR13/50 and pooled plasma from 4 mice (5–6 hr after food removal in the morning). A total of 45–0.5 ml samples were collected from each fractionation. Cholesterol concentration was measured as described above, and lipoprotein apoA-I and apoA-IV distribution was determined by Western blot analysis as described below.

### Lipoprotein isolation and labeling

Plasma from human donors was subjected to serial gradient ultracentrifugation as described[18]. LDL was isolated from the density range 1.019 to 1.063 g/ml, HDL_2_ from 1.063–1.125 g/ml and HDL_3_ from 1.125–1.210 g/ml. Isolated lipoproteins were dialyzed against 4 L of 0.8% saline-EDTA overnight at 4°C under nitrogen gas. Protein concentration was determined using the BCA assay (Bio-Rad, Hercules, CA).

Acetylated-LDL (ac-LDL) were prepared and labeled as described[19]. Briefly, LDL isolated from human plasma (0.5–1.0 mg protein/ml) was diluted 1∶1 with saturated sodium acetate while continuously stirred on ice. Acetic anhydride was added with stirring in aliquots (2 µl) and up to 1.5 times the protein mass (1.5 µl/µg). The mixture was stirred for 30 more min then dialyzed (24–36 h, 4°C) against 2 liters of buffer (0.15 M NaCl, 0.3 mM EDTA, pH 7.4) with 6 changes (total 12 L). Protein content was determined after dialysis. LDL or ac-LDL (1 ml, 0.5 mg protein) were labelled [20] either with^ 3^H-cholesterol-oleate (50 µCi, Perkin Elmer Life Sciences) or ^14^C-cholesterol (50 µCi, American Radiolabeled Chemicals, St. Louis, MO).

For HDL clearance assay, human HDL_3_ were dual labeled with ^125^I-dilactitol tyramine and ^3^H-cholesteryl oleolyl ether [9], [21]. The ^125^I/^3^H-hHDL_3_ was dialyzed against buffer (0.15 M NaCl, 0.3 mM EDTA) to remove free label. Specific activity was 20.8±0.1 (^125^I) and 6.4±0.3 (^3^H) dpm/ng protein.

### *In vivo* HDL clearance

Anesthetized mice (5 females and 10 males per genotype) were injected 0.2-ml of labeled hHDL_3_ (∼180 µg hHDL_3_ protein, 1.7 µCi ^125^I and 0.5 µCi ^3^H) via the external jugular vein [22], [23]. Venous blood was collected from the retro-orbital plexus in heparin-coated capillaries (∼50 µl) at 1, 5, 10, 20, 30 40, 60, 90, 120, 180, and 240 minutes later. Radioactivity (^3^H and ^125^I) was counted in plasma and normalized to HDL concentration. Data are expressed as percent of levels at 1 min after injection.

### *In vitro* HDL and LDL uptake by primary hepatocytes

Mouse hepatocytes were isolated [24] and maintained in DMEM with 10% FBS. For HDL assays, dual-labeled HDL_3_ were added to the media (0.1, 0.2, and 0.3 mg/ml HDL_3_) for 3 hr. The medium was then removed and the cells washed with PBS before lysis in 0.2N NaOH for radioactivity counts, later adjusted for protein content. For LDL assays, hepatocytes were incubated with ^3^H-cholesteryl oleolyl labeled hLDL (0.1 mg/ml) for 5 hr before processing as for HDL uptake.

### ApoA-I and apoA-IV synthesis and secretion by primary hepatocytes

Synthesis and secretion of apoA-I and apoA-IV by primary hepatocytes were determined using ^35^S labeled methionine and cysteine[25]. Freshly isolated hepatocytes were incubated in methionine and cysteine-free DMEM (Sigma Aldrich, St. Louis, MO) for 30 minute then switched to the same medium but with 5 µCi/ml ^35^S labeled Met and Cys (Easytag Protein Labeling mix, Perkin Elmer, Waltham, MA) for 3 hr. Medium and cells were harvested, and immunoprecipitated again apoA-I, apoA-IV, and albumin antibodies. Proteins were separated by SDS-PAGE, fixed, dried, and exposed against X-ray film. Albumin abundance was used as an internal standard.

### Cholesterol and phospholipid efflux assays in primary hepatocytes and macrophages

Mice were intraperitoneally injected with 2 ml of 4.5% thioglycollate 4 days before harvesting peritoneal macrophages by lavage[26]. The macrophages were incubated overnight in 12-well plates (0.5×10^6^ cells/well) in RPMI-1640 supplemented with 10% FBS and antibiotics.

Cholesterol and phospholipid efflux assays were as described [20], [27]. For cholesterol efflux, the cells were loaded with cholesterol for 18 hr in DMEM (hepatocyte) or RPMI-1640 (macrophage) with 0.2% FA-free BSA, labeled LDL or ac-LDL (1 µCi/well) and ACAT inhibitor (10 nM oleic acid-2,6-diisopropylanilide, Cayman Chemical Co., Ann Arbor, MI). The next day, the cells were washed twice with pre-warmed PBS and incubated in fresh DMEM- or RPMI-0.2% BSA for 15 min before switching to medium containing isolated hHDL_2_ or purified human apoA-I (A0722, Sigma-Aldrich, St. Louis, MO) at the indicated concentrations for a 12 hr efflux assay. Media were collected and cleared from cell debris by centrifugation. Cells were washed and scrapped in cold PBS with proteinase inhibitor (Sigma-Aldrich, St. Louis, MO). For phospholipid efflux assays, cellular phospholipids were labeled by incubating the cells with 0.5 µCi [^3^H]-choline (American Radiolabeled Chemicals, St. Louis, MO) in DMEM or RPMI-10% FBS for 24 hr. After washing the cells were incubated in serum-free medium with hHDL_2_ for 12 hr. Media and cells were then extracted for lipids (chloroform/methanol, 1∶1, v/v). Lipid radioactivity was counted and adjusted for cellular protein content.

### Biotin labeling of cell membrane proteins

For biotin labeling of surface proteins[28] cells were incubated in PBS (with 0.1 mM Ca^2+^, 0.5 mM Mg^2+^) with 500 µg/ml sulfo-succinimido-biotin (Pierce-Thermo Scientific, Waltham, MA) for 30 min at 4°C. The washed cells were lysed (50 mM Tris, 1 mM EDTA, 1%Triton, 2%SDS, 1X proteinase inhibitor, pH = 7.4) and about 10% of lysates was saved for protein assays. The rest was used for immunoprecipitation of biotin-labeled proteins using streptavidin-magnetic beads (2 hrs, 4°C). Biotin-labeled proteins were separated by SDS-PAGE alongside total cell proteins. Antibodies for Cadherin 1(CDH1, Abcam, Cambridge, MA) was used as a cell membrane marker.

### RT-PCR

Total liver RNA was isolated using Trizol (Invitrogen, Carlsbad, CA) and further purified by RNeasy columns (Qiagen Sciences, Valencia, CA). cDNA was synthesized using high capacity cDNA reverse transcription kit (Applied biosystems, Foster City, CA) and changes in transcript abundance were measured using SYBR-green PCR Master kit on a ABI-7500 real-time PCR system. For each genotype, mRNA from 8 mice was randomly combined to form 2 pools of equal concentration. Each primer pair was repeated 3 times on a 96-wells plate (6 measurements per genotype) and duplicate plates were used. The *36B4* gene was used as a normalization standard. The RT-PCR primer sequences and relevant references were listed in Table S1.

### Western Blots

Proteins separated by SDS-PAGE were transferred to a PVDF membrane (Millipore, St. Charles, MO) and probed with antibodies. Polyclonal antibodies against mouse apoB, apoA-I, apoA-IV, and apoE were generated and used as reported[24]. Rabbit polyclonal antibody against mouse SR-B1 (Novus Biologicals, Litteton, CO), mouse monoclonal antibodies against ABCA1, ABCG1, CDH1(Abcam, Cambridge, MA), PPARγ and β-Actin (Santa Cruz Biotech, CA) were used as recommended by the manufacturers. Immune signals were detected and quantified using LI-COR Odyssey Imaging (Lincoln, NE) with near-infrared dye labeled secondary antibodies except for ^35^S labeling of hepatic apolipoprotein synthesis and secretion where X-ray film was used.

### Statistical Analysis

Statistical analyses used SAS (9.02 Cary, NC). Differences were considered significant if the p-value was less than 0.05.

## Results

### HDL levels and distribution in CD36^−/−^ and WT mice fed a high cholesterol diet

**Figure 1:**
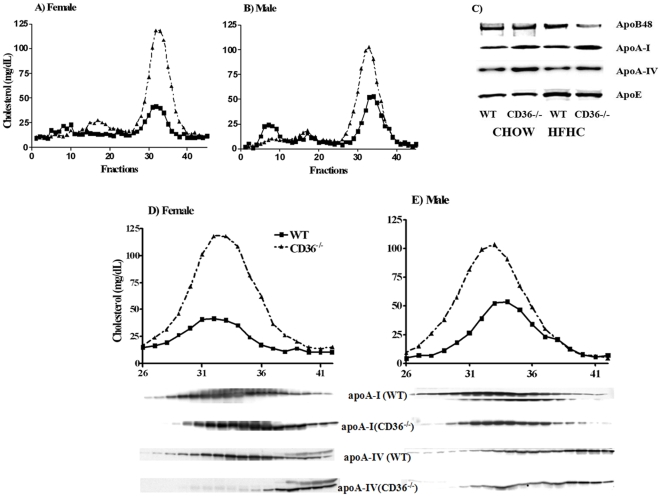
Effect of a cholesterol rich diet on plasma cholesterol and apolipoprotein content of HDL subfractions in WT and CD36 null (CD36^−/−^) mice. Pooled plasma (4–5 mice per genotype) collected in the postprandial state (5–6 hrs after food removal) was used for fractionation. **Panels A and B:** Cholesterol content of FPLC fractions, WT (solid line), CD36^−/−^ (dashed line), female (A), male (B). **Panel C:** Western blot of plasma apolipoproteins from mice fed CHOW or high fat high cholesterol (HFHC). **Panels D and E:** Expanded graphs showing cholesterol in HDL subfractions 26–42 and the corresponding apolipoprotein distribution. HDL fractions were separated by SDS-PAGE and blotted against polyclonal antibodies for apoA-I and apoA-IV. Data are representative of two fractionation assays per experiment from two separate experiments.

The initial report on CD36 deficient mice fed a chow diet with 4% fat described a modest increase (30%) of plasma cholesterol and HDL as compared to WT mice[11] but increased HDL were not noted in other studies[5], [29]. As shown in Table 1, we confirmed observations[11] of significantly higher plasma cholesterol in CD36^−/−^ as compared to WT mice maintained on chow and the difference was observed in both males and females. Phospholipid (PL) levels were also increased in female but not in male CD36^−/−^ mice. To further validate the differences in HDL levels between the genotypes, the mice were challenged with a high fat, high cholesterol (HFHC) diet for 4 weeks. The HFHC diet significantly increased blood cholesterol and PL (Table 1) in all mice. However, levels of both lipids were significantly higher (1.5–2 fold) in CD36^−/−^, as compared to WT mice, regardless of sex. The increased serum cholesterol was primarily localized to HDL subfractions as shown in Figure 1(A and B). Consistent with the higher HDL concentrations, plasma levels of HDL constituent apolipoproteins, apoA-I and apoA-IV, were also higher in CD36^−/−^mice (Figure 1C). On the other hand, levels of apoB48 and apoE (components of chylomicrons and VLDL) were similar. Figure 1C also shows that the apolipoprotein profile was not altered by the diet except for a HFHC-induced increase in apoE levels. These data showed that CD36^−/−^ mice exhibit higher levels of plasma cholesterol than WT mice and this difference is exaggerated upon feeding a cholesterol rich diet. The higher HDL levels were present in male and female mice and were not altered by the nutritional state of the mice; HDL levels, in fed versus 6 and 14 hr fasted mice were reproducibly higher in CD36^−/−^ as compared to WT mice (data not shown).

**Table 1:** Serum phospholipids and cholesterol in WT and CD36null mice fed chow or high fat high cholesterol diets for 4 weeks.

	Gender	WT	CD36^−/−^	P value
		**CHOW**		
**Chol (mg/dL)**	F	83.0±7.4	132.3±18.2	**0.001**
	M	83.2±7.0	111.5±15.7	**0.003**
**PL (mg/dL)**	F	62.6±7.5	135.3±7.7	**<0.001**
	M	95.3±7.8	73.3±32.8	0.196
		**HFHC**		
**Chol (mg/dL)**	F	128.4±31.1	194.7±23.3	**<0.001**
	M	138.6±51.2	236.8±56.8	**<0.001**
**PL (mg/dL)**	F	87.0±18.2	185.5±22	**<0.001**
	M	126.9±32.4	186.0±42.9	**0.035**

**1. Parameters are from 12-week old mice after an overnight fast, n = 8 per genotype.**

**2. P value is for differences between WT and CD36^−/−^ mice.**

**3. Chol, cholesterol; PL, phospholipids.**

We next examined whether there were changes in apolipoprotein content of HDL subfractions collected from FPLC. As shown in Figure 1D and E, apoA-I and apoA-IV distribution in CD36^−/−^ samples shifted to higher density fractions (more apparent in female mice) suggesting presence of more lipid poor HDL particles.

### *In vivo* HDL clearance

**Figure 2:**
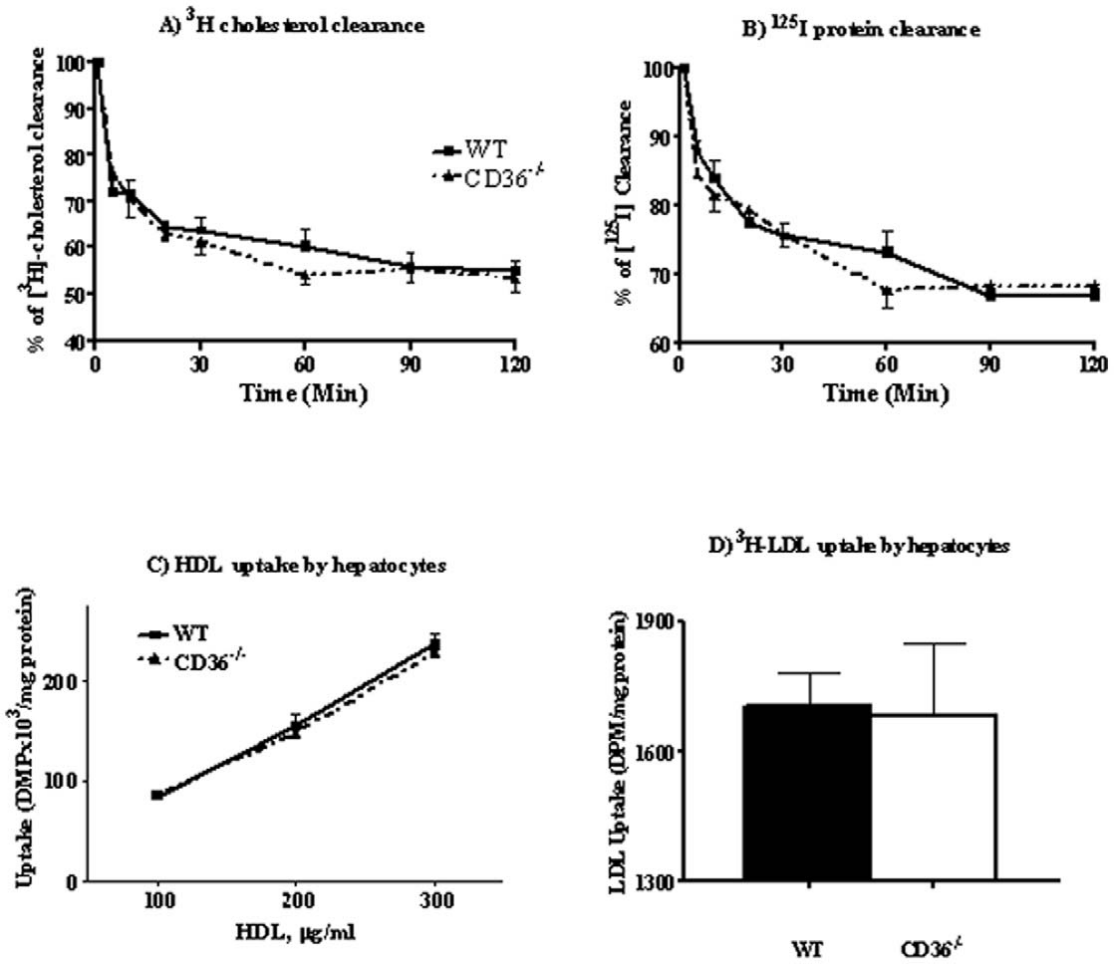
HDL clearance *in vivo* and cholesterol uptake by isolated hepatocytes *in vitro*. Panels A and B: *In vivo* HDL clearance. 200 µl of [^3^H]- and [^125^I]-dual labeled human HDL was injected into the mouse tail vein and plasma radioactivity (**panel A:** [^3^H] and **panel B:** [^125^I]) was measured at the times indicated. Five females and 10 males were used per genotype. **Panels C and D:** *In vitro* cholesterol uptake. Hepatocytes isolated from WT and CD36^−/−^ mice, were plated at a density of 0.5×10^6^ cells/well and assayed for cholesterol uptake, using either HDL or LDL. **Panel C:** Cholesterol uptake from HDL using three concentrations of ^3^H-cholesteryl oleolyl ether labeled human HDL_3_ for 3 hours. **Panel D:** Cholesterol uptake from LDL with cells incubated with ^3^H-cholesteryl oleolyl labeled native hLDL for 5 hr. Data are means (n = 4) shown with standard errors and representative of two separate experiments.

A major regulator of plasma HDL levels is HDL clearance by hepatocyte-mediated selective CE uptake[30]. Adenoviral overexpression of CD36 in the liver was previously found not to alter plasma HDL[10]. We examined whether CD36 deletion, which alters plasma HDL, impacts HDL clearance by reducing hepatic CE uptake[9]. CD36 deficiency could also impact the process indirectly through altered activation of peroxisome proliferator-activated receptors (PPARs)[31] which modulate several aspects of lipoprotein metabolism[32]. We quantified *in vivo* HDL catabolism using ^125^I (apolipoprotein) and ^3^H-cholesteryl oleolyl ether dual-labeled human HDL_3_. Each mouse was injected with 200 µl of labeled lipoprotein and plasma radioactivity was measured over 2 hrs. Counts were adjusted for the higher HDL levels in CD36^−/−^ mice. As shown in Figure 2, there was no difference in HDL-CE clearance rate between genotypes (Fig. 2A) and clearance of ^125^I-HDL apolipoprotein was also similar (Fig. 2B). This suggested that HDL clearance does not contribute to elevating plasma HDL in CD36^−/−^ mice.

### HDL and LDL uptake by isolated hepatocytes

To further validate the interpretation that hepatic HDL clearance is not altered in CD36^−/−^ mice, we compared directly *in vitro* selective CE uptake by primary hepatocytes isolated from WT and CD36^−/−^ mice. The cells were incubated with different concentrations (0.1, 0.2, and 0.3 mg/ml) of [^3^H]-cholesteryl oleolyl ether labeled hHDL_3_ for 3 hrs. Selective cholesterol uptake showed an almost linear response to hHDL_3_ loading and was similar for cells from WT and CD36^−/−^ mice as shown in Figure 2C.

Cholesteryl ester in HDL can be transferred to apoB-containing lipoproteins and taken up by the liver via the LDL receptor[33]. The possibility that this pathway is enhanced in CD36^−/−^ hepatocytes was tested using ^3^H-cholesterol oleate labeled hLDL and as shown in Figure 2D, no genotype related difference was observed. The above data indicated that hepatocyte handling of CE from HDL or LDL was unaltered by CD36 deficiency.

### Efflux of cholesterol and phospholipids from primary hepatocytes

**Figure 3:**
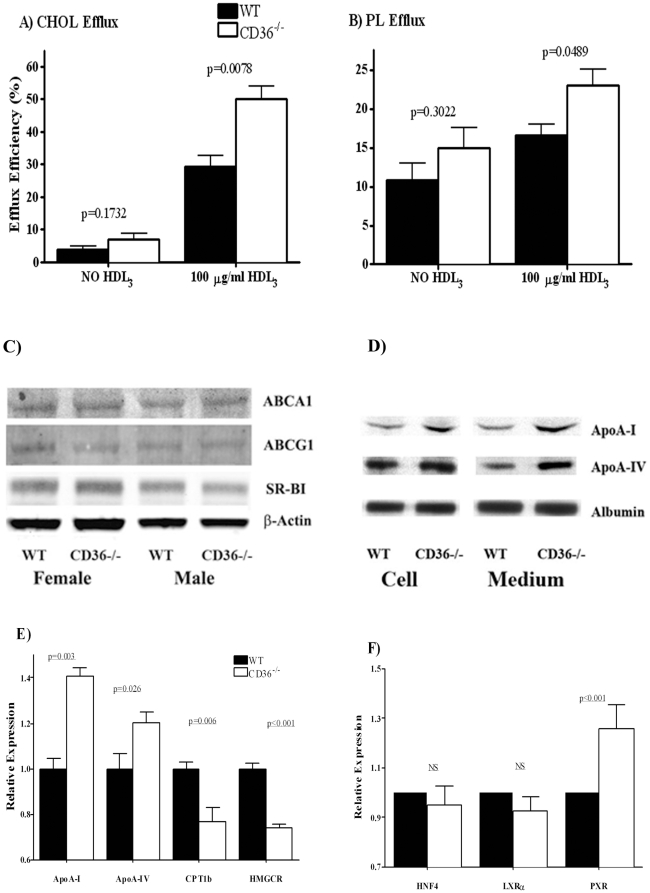
Hepatocytes from WT and CD36^−/−^ mice. Cholesterol and phospholipid efflux, apoA-I production and expression of genes that regulate cholesterol metabolism. Primary hepatocytes were isolated from WT and CD36^−/−^ mice and assayed for cholesterol and phospholipid efflux. Hepatocytes were loaded with ^14^C-cholesterol and ^3^H-choline chloride (to label cell phospholipids) as described in Methods. Cells were then allowed to efflux ^14^C-cholesterol and ^3^H-phosphotidylcholine overnight. **Panel A:** cholesterol efflux and **Panel B:** phospholipid efflux for WT and CD36^−/−^ hepatocytes. Data (DPM adjusted for cellular protein) are means (n = 4) shown with standard errors and typical of three experiments. **Panel C:** Western blot showing expression of proteins related to cholesterol and phospholipid efflux. Data are representative of three separate experiments. **Panel D:** ApoA-I production and secretion assayed in primary hepatocytes labeled with ^35^S. Data are from two separate experiments with 3 independent samples in each experiment. Relative band intensity (intensity of apoA-I/intensity of albumin band) was quantified using ImageJ software (http://rsbweb.nih.gov/ij/). ApoA-I levels were significantly higher in CD36^−/−^ as compared to WT hepatocytes (p = 0.011 and 0.002 for cell and media respectively). ApoA-IV secretion was significantly higher in CD36^−/−^ (p = 0.008), but not production (p>0.05). **Panel E:** Gene expression measured by RT-PCR of transcriptional factors that regulate apoA-I expression and **Panel F:** of genes targeted by PXR. In both panel E and F, n = 6 on each assay plate, and data are representative of three separate experiments.

The possibility that there was alteration of cholesterol efflux by the CD36^−/−^ liver was tested next. Cholesterol efflux from hepatocytes can influence plasma HDL concentrations. In addition efflux of cholesterol especially that mediated by the ABCA1 transporter is coupled to that of phospholipids[6]. As shown earlier in Table 1, differences in both plasma cholesterol and phospholipid levels were measured in CD36^−/−^ as compared to WT mice. We examined efflux efficiency of these lipids by primary hepatocytes isolated from both mice groups. Freshly isolated hepatocytes were first loaded with ^14^C-cholesterol or with ^3^H-choline chloride (to label cell phospholipids). Cells were then allowed to efflux ^14^C-cholesterol or ^3^H-phosphotidylcholine to HDL_3_ 0.1 mg/ml overnight. Surprisingly, as shown in Figure 3A and B, CD36^−/−^ hepatocytes had significantly higher cholesterol and phospholipid efflux efficiency. At the end of the incubation with HDL_3_, about 30% of labeled cholesterol effluxed into the medium in WT hepatocytes as compared to 50% for CD36^−/−^ cells (Fig. 3A). CD36^−/−^ cells also effluxed a higher proportion of cellular PL than WT cells (Fig. 3B). These data suggested that CD36 may negatively influence hepatic cholesterol and phospholipid efflux so this is enhanced in CD36 deficiency.

Cholesterol and phospholipid efflux from hepatocytes is primarily facilitated by the ATP cassette transporters ABCA1 and ABCG1[34] with a more minor role for SR-B1[35]. We examined whether increased cholesterol efflux was due to increases in these proteins. However, Western analysis using liver homogenates showed no differences between genotypes (Fig. 3C).

### Synthesis and secretion of ApoA-I and IV by isolated hepatocytes

Elevated HDL can result from over-production of apoA-I[36]. In addition as shown in Figure 1, CD36^−/−^ mice had higher plasma levels of HDL apolipoproteins, so we examined whether CD36 deficiency alters hepatic apoA-I and apoA-IV production and secretion. Hepatocytes from WT and CD36^−/−^ mice were labeled with ^35^S for 3 hr then cellular and medium apoA-I and apoA-IV were immunoprecipitated using the respective antibodies. Figure 3D shows that apoA-I and apoA-IV synthesis and secretion were increased in CD36^−/−^ as compared to WT cells (p = 0.011 and 0.002 for cellular and media apoA-I content respectively). This suggested that enhanced production of HDL apolipoproteins may contribute to the high HDL in CD36^−/−^ mice.

To determine whether the increased synthesis of apoA-I and apoA-IV in CD36^−/−^ hepatocytes involved transcriptional or post-translational mechanisms, we measured hepatic mRNA of the corresponding genes. Levels of *APOA-I* and *APOA-IV* mRNA were significantly higher in CD36^−/−^ livers (Fig. 3E). Hepatic expression of apoA-I and apoA-IV is regulated by many factors, including pregnane X receptor (PXR) and hepatocyte nuclear factor 4 (HNF4)[37]. RT-PCR showed that PXR expression in CD36^−/−^ liver was significantly increased while that of HFN4 and LXR was unaltered (Fig. 3F). PXR represses transcription of genes involved in lipid β-oxidation, such as carnitine palmitoyl transferase 1(*CPT1*) and 3-Hydroxy-3-Methylglutaryl CoA Reductase (*HMGCR*)[38]. Consistent with enhanced PXR expression, hepatic *CPT1b* and *HMGCR* expression was significantly lower in CD36^−/−^ as compared to WT mice (Fig. 3E). Expression of ABCA1 was slightly increased while that of ABCG1 was not altered.

Overall, the findings indicated that CD36^−/−^ mice have increased hepatic apoA-I and apoA-IV synthesis and secretion as well as enhanced hepatocyte cholesterol and phospholipid efflux. Both changes would contribute to elevating plasma HDL in CD36 deficiency.

### Efflux of cholesterol and phospholipids from isolated macrophages

A cell type that is central to cholesterol homeostasis is the macrophage. Conversion of macrophages to lipid laden foam cells as a result of excess cholesterol uptake or defective cholesterol efflux is implicated in lesion development[6]. CD36 deficiency reduces uptake of modified lipoproteins by macrophages limiting foam cell formation[2]. We examined whether the deficiency also influences macrophage cholesterol efflux similar to its effect on hepatocytes. Intraperitoneal macrophages from WT and CD36^−/−^ mice were assayed for capacity to efflux CE to HDL. In a first approach, macrophages were preloaded overnight with [^3^H]-cholesterol oleate labeled ac-LDL (1.0×10^6^ dpm/mg protein). The washed cells were switched to serum-free RPMI containing 0.1 mg/ml hHDL_2_ for 12 hrs and then cells and media were collected for [^3^H] counts.

**Figure 4:**
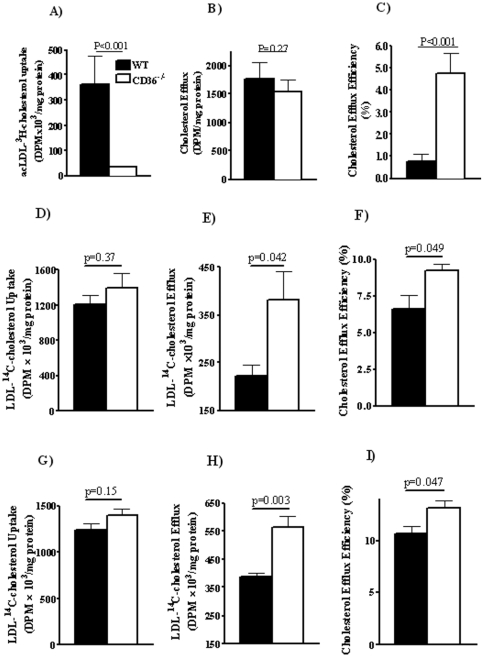
Cholesterol efflux in macrophages from WT and CD36−/− mice using different lipoprotein substrates. Macrophages isolated from WT and CD36^−/−^ mice were cholesterol loaded by preincubation (12 hr) with ^3^H-cholesterol oleate labeled ac-LDL (**panels A, B and C**) or ^14^C-cholesterol labeled native LDL (**panels D, E, F, G, H, and I**). For cholesterol efflux assays, the cells were switched to a serum-free medium containing 0.1 mg/ml human HDL_2_- for 8 hours. Media and cells were then collected and assayed for radioactivity. **Panel A:** Cholesterol uptake from ac-LDL by CD36^+/+^ and CD36^−/−^ macrophages. **Panel B:** Cholesterol efflux into HDL-containing medium. **Panel C:** Efflux efficiency, ^3^H-cholesterol oleate efflux expressed as percent of cellular ^3^H at efflux initiation. Data are means (n = 4) shown with standard errors and are typical of three separate experiments. Panel D to F: macrophage efflux assay using hHDL_2_ as efflux substrate. **Panel D:** [^14^C]-cholesterol uptake by macrophages preincubated with native ^14^C-LDL (0.3 mg/ml). **Panel E:** Cholesterol efflux by 50 µg/ml HDL_2_ (DPM adjusted for cell protein). **Panel F:** Efflux efficiency (percent of total ^14^C in the media adjusted by protein content). Data are means with standard errors (n = 4) typical of three separate experiments. Panel G to I: macrophage efflux assay using human apoA-I as efflux substrate. **Panel G:** [^14^C]-cholesterol uptake by macrophages preincubated with native ^14^C-LDL (0.3 mg/ml). **Panel H:** Cholesterol efflux by 50 µg/ml human apoA-I (DPM adjusted for cell protein). **Panel I:** Efflux efficiency (percent of total ^14^C counts in the media adjusted by protein content). Data are means with standard errors (n = 4).

CD36^−/−^ macrophages demonstrated the expected impairment of ac-LDL cholesterol uptake[5] accumulating about 25% of counts recovered in WT cells (Fig. 4A). However, despite the lower level of cholesterol loaded into CD36^−/−^ cells, a similar amount of [^3^H]-cholesterol effluxed into the HDL containing medium (Fig. 4B). Percent cholesterol efflux ([^3^H] in media/total [^3^H] adjusted by protein content) for CD36^−/−^ macrophages was about 4–6% as compared to ∼1% for WT cells (Fig. 4C). To separate cholesterol uptake and efflux we equalized cellular cholesterol loading before efflux initiation by incubating macrophages with unmodified LDL labeled with [^14^C]-cholesterol. LDL uptake by macrophage is mediated by the LDL receptor[5], [39] independent of CD36 so [^14^C]-LDL loading resulted in similar [^14^C]-cholesterol accumulation by WT and CD36^−/−^ macrophages (p = 0.37, Fig. 4D). Again, as shown in Figure 4E, cells from CD36^−/−^ mice effluxed more [^14^C]-cholesterol into the medium (p = 0.042), and had significantly higher efflux efficiency (p = 0.049, Fig. 4F). Thus the enhanced cholesterol efflux of CD36^−/−^ cells was independent of cellular cholesterol.

Some studies suggested that cholesterol efflux mediated by ABCA1 favors lipid-poor apoA-I instead of HDL particles[6]. We also conducted efflux experiments using 50 µg/ml human apoA-I as the lipid acceptor. As shown in Figure 4G–H, cells from CD36^−/−^ mice effluxed more cholesterol to media apoA-I (p = 0.003, Figure 4H) and had higher efflux efficiency than WT cells (p = 0.047, Figure 4I).

**Figure 5:**
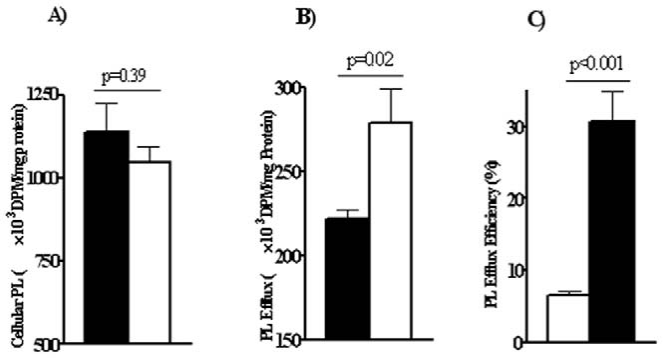
Phospholipid efflux in macrophages from WT and CD36−/− mice. **Panel A:** Content of ^3^H-labeled phospholipids in macrophages preincubated with 0.5 µCi/ml of [^3^H]-choline chloride for 24 hours. Panel B: PL efflux (DPM/cell protein) into serum-free RPMI with hHDL_2_ for 8 hr. Panel C: Efflux efficiency. Data are means (n = 4) shown with standard errors and are typical of three separate experiments.

To examine PL efflux, macrophages were preincubated with [^3^H]-choline (24 hrs), washed and switched to medium containing 0.2 mg/ml human HDL_2_ for 8 hrs. Radioactivity was determined in lipid extracts of cells and medium. Figure 5A shows that [^3^H] incorporation into cellular PL was similar for WT and CD36^−/−^ macrophages. However, as with cholesterol, CD36^−/−^ macrophages showed enhanced PL efflux (Fig. 5B), and efflux efficiency (Fig. 5C).

**Figure 6:**
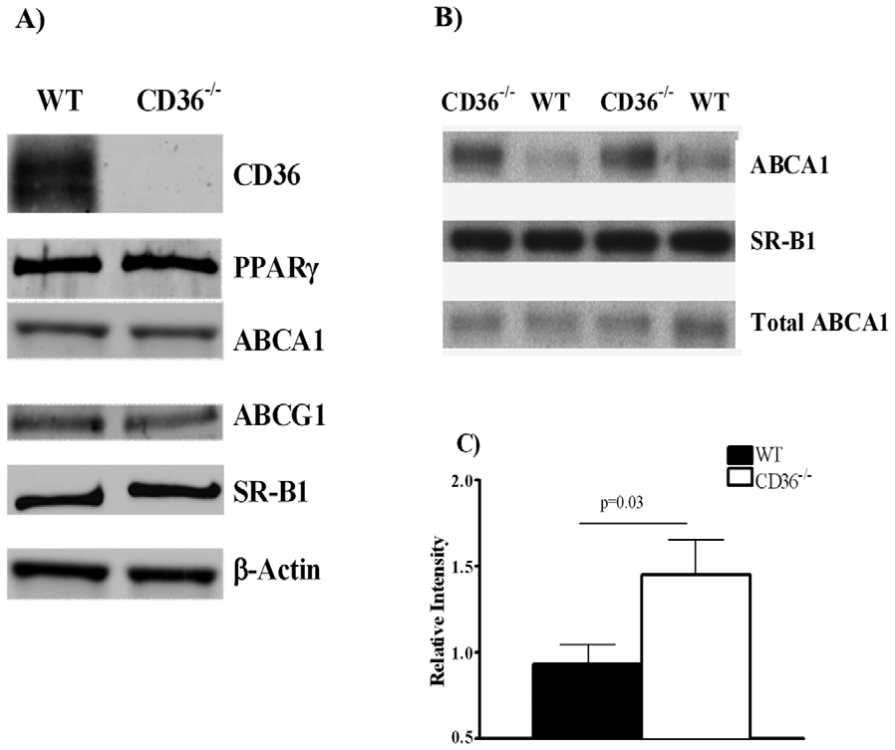
Total and cell surface proteins in primary macrophages from WT and CD36^−/−^ mice. **Panel A**. Total protein content, determined by western blot for macrophages isolated and pooled from 4–5 mice per genotype. Detection was by infrared secondary antibodies. **Panel B**: Cell-surface proteins determined using biotin labeling as described in Methods (first row). CDH1 was used as a cell surface protein marker (the second row). Total cellular ABCA1 for respective samples was listed in the third row. **Panel C.** Relative intensities of the macrophages surface proteins ABCA1 between WT and CD36^−/−^ mice. Intensity of protein bands (ABCA1) from 8 independent samples (4 WT and 4 CD36^−/−^) were quantified and expressed as a relative intensity of intensity of CDH1 in the same sample.

Cholesterol and phospholipid efflux from macrophage is primarily facilitated by ABCA1 and to a lesser extent by ABCG1[34] and SR-B1[35]. ABCA1 expression in macrophages is stimulated by liver-X-receptors (LXRs) which in turn is modulated by peroxisome proliferator-activated receptor gamma (PPARγ)[30]. We examined whether CD36 deficiency associates with alterations in protein levels of PPARγ, ABCA1, ABCG1 and SR-B1. As Figure 6A shows, there were no noticeable differences in the amount of these proteins between WT and CD36^−/−^ macrophages. ABCA1 recycles between plasma membrane and intracellular compartments and membrane transporter content can be altered without changes in total ABCA1 protein[40]. We determined whether CD36 deficiency alters ABCA1 membrane content by biotin labeling of macrophage surface proteins. As shown in Figure 6B, these assays documented a significant increase of cell surface ABCA1 in CD36^−/−^ as compared to WT macrophages. In contrast, cell-surface SR-B1 was minimally altered. When cell surface ABCA1 was adjusted by cell membrane marker Cadherin 1 (CDH1), membrane-bound ABCA1 in CD36^−/−^ cells was almost double that in WT cells (p = 0.03, Figure 6C). These data suggested that CD36 increases cholesterol efflux in macrophages at least in part by influencing cell membrane ABCA1 distribution. A similar increase in surface ABCA1 was observed in CD36^−/−^ hepatocytes suggesting that this interpretation may also apply to cholesterol efflux from these cells (Figure S1). Although preparations of isolated hepatocytes are largely pure as a result of the fact that hepatocytes are the major cell type in the liver, he possibility that the increase in cholesterol efflux from hepatocytes could reflect contaminating macrophages was ruled out by assaying expression of the macrophage specific marker F4/80. As shown in Figure S2, F4/80 levels in the hepatocyte preparations were very low to undetectable.

## Discussion

There is strong evidence in rodent models and humans to support a role of CD36 in uptake of FA and oxidized lipoproteins and in mediating signal transduction in response to these ligands[1], [2]. CD36 is also a high affinity receptor for native lipoproteins as reported in 1998[8]. However, its role in the metabolism of these particles has been relatively little studied. Recent work documented that CD36 can facilitate CE uptake from native LDL and that it delays LDL clearance *in vivo* [5]. In the case of HDL, CD36 binds these particles as well as SR-B1 but is less efficient in mediating selective HDL-CE uptake and does not promote whole particle endocytosis[9], [10], [41], [42], [43]. *In vivo* CD36 overexpression in the liver did not alter plasma HDL in mice so the functional relevance of the protein in HDL metabolism was considered minimal[10]. Our recent family based association study in a large African American population sample, identified a surprising number of CD36 SNPs that influenced plasma HDL[17]. These findings, together with the earlier observation of high cholesterol and HDL levels in CD36 knockout mice[11] led us to consider a potential physiological role of CD36 in HDL metabolism, which was explored in the present study. HDL distribution and kinetics in CD36^−/−^ mice and impact of CD36 deficiency on cholesterol uptake and efflux in primary hepatocytes and macrophages were examined. The findings document that CD36 influences plasma apoA-I, apoA-IV, cholesterol and phospholipid levels. The mechanisms involved include increased hepatic expression and synthesis of HDL apolipoproteins and enhanced efflux of cholesterol and phospholipids by hepatocytes and macrophages. The data demonstrate a novel role of CD36 in the regulation of reverse cholesterol transport.

CD36 null mice, regardless of sex, exhibited an exaggerated response of plasma HDL to increased dietary supply of cholesterol. HDL levels were substantially higher in CD36^−/−^ mice and parallel increases were measured in plasma phospholipids and cholesterol. HDL clearance in mice is mainly accomplished via selective CE uptake by the liver primarily via SR-B1 (reviewed in [7], [44]). Reduced activity of hepatic SR-B1 impedes HDL clearance and increases serum HDL levels [45], [46], [47]. CD36 expression in the liver is relatively low but levels are up-regulated under various conditions associated with steatosis[5], [48]. The low hepatic CD36 levels, the previous demonstration that CD36 is less efficient than SR-B1 in selective HDL-CE uptake, and the finding that hepatic CD36 overexpression in mice did not alter plasma HDL levels[10] suggested that CD36 function in HDL clearance may not be relevant physiologically[9], [10]. Our findings using the opposite condition of CD36 deletion also demonstrate that *in vivo* the protein does not significantly contribute to HDL clearance. Absence of CD36 did not alter kinetics of labeled HDL *in vivo* and CD36 deficient hepatocytes assayed *in vitro* exhibited rates of HDL uptake that were similar to CD36 sufficient hepatocytes. Thus defective HDL clearance is not responsible for the elevated HDL in CD36^−/−^ mice or for the exaggerated response of plasma cholesterol and phospholipids in these mice to the high cholesterol diet.

The mechanisms underlying the effect of CD36 deficiency on HDL levels involve a combination of increased hepatic apoA production and enhanced cellular efflux of cholesterol and phospholipids. ApoA-I is the major HDL apolipoprotein and is mainly synthesized in the liver[37]. ApoA-I regulation is transcriptional[37] or post-transcriptional[49]. ApoA-I expression is influenced by PPARα, PXR and HNF4. The increase in apoA synthesis in livers of CD36^−/−^ deficient mice may reflect a PXR-mediated increase in apoA-I gene transcription. Like apoA-I, CD36 is a direct target of PXR[48] and the increased PXR expression in the CD36^−/−^ liver suggests a regulatory loop similar to the one that exists between CD36 and PPARδ[31].

The enhanced apoA supply in CD36^−/−^ mice would promote reverse cholesterol transport *in vivo* but it does not explain the enhanced efflux of cholesterol that is observed in isolated CD36^−/−^ hepatocytes and macrophages *in vitro*. The elevated cholesterol/PL efflux in CD36^−/−^ cells appears due at least in part to enhanced cell surface ABCA1 re-distribution. ABCA1 is present in the plasma membrane as well as in subcellular compartments such as late endosomes and lysosomes[50]. Internalization and recycling of ABCA1 are integral to cholesterol mobilization, which involves cholesterol pools in the membrane, the endoplasmic reticulum in addition to endocytic and lysosomal compartments[28], [50]. CD36 may influence ABCA1 recycling directly or via altering distribution of cholesterol and phospholipids in intracellular pools related to apoA-I or nascent HDL lipidation. Recent evidence points to the important role of lipid droplets in distributing various lipids to membrane-bound intracellular organelles[51], [52], [53]. CD36 is among proteins identified on lipid droplets by proteomic studies [[54] and N Wolins, personal communication] but whether it plays a role in droplet formation is unknown. In human carotid lesion samples, CD36 expression positively correlates with adipophilin (ADRP)[55], a lipid droplet protein that also influences cellular cholesterol distribution[56] and efflux[57]. ADRP protein levels are significantly reduced in CD36^−/−^ macrophages (Fig. S3). Thus CD36 may impact ABCA1 localization and cholesterol efflux via influencing intracellular lipid partitioning, which would be consistent with its effect on lipoprotein production in the intestine[14] and possibly the liver (Fig. 3). ABCA1 trafficking involves rab proteins[40] and the possibility that these may be altered in CD36^−/−^ cells will also need to be considered.

Overall the findings both *in vivo* and in primary cells *in vitro* support a negative relationship between CD36 expression and HDL formation. They provide potential mechanisms for the associations observed in humans between polymorphisms in the *CD36* gene and plasma HDL[17]. A coding variant that results in partial CD36 protein deficiency associated with higher HDL levels and was protective against the metabolic syndrome. Furthermore, our unpublished findings in humans [Love-Gregory et al] indicate that monocyte CD36 level is inversely related to plasma HDL. Thus it is possible that an important influence of CD36 *in vivo* is on cholesterol efflux and reverse cholesterol transport. CD36 downregulation may be beneficial for protection from atherosclerotic disease as it would reduce foam cell formation via enhancing cholesterol efflux in addition to reducing cholesterol uptake by macrophages.

## Supporting Information

Table S1 (0.04 MB DOC)

Figure S1 (0.05 MB DOC)

Figure S2 (0.03 MB DOC)

Figure S3 (0.10 MB DOC)

## References

[1] Su X, Abumrad NA (2009). Cellular fatty acid uptake: a pathway under construction.. Trends Endocrinol Metab.

[2] Silverstein RL, Febbraio M (2009). CD36, a scavenger receptor involved in immunity, metabolism, angiogenesis, and behavior.. Sci Signal.

[3] Nicholson AC, Febbraio M, Han J, Silverstein RL, Hajjar DP (2000). CD36 in atherosclerosis. The role of a class B macrophage scavenger receptor.. Ann N Y Acad Sci.

[4] Zeng Y, Tao N, Chung KN, Heuser JE, Lublin DM (2003). Endocytosis of oxidized low density lipoprotein through scavenger receptor CD36 utilizes a lipid raft pathway that does not require caveolin-1.. J Biol Chem.

[5] Luangrath V, Brodeur MR, Rhainds D, Brissette L (2008). Mouse CD36 has opposite effects on LDL and oxidized LDL metabolism in vivo.. Arterioscler Thromb Vasc Biol.

[6] Tall AR, Yvan-Charvet L, Terasaka N, Pagler T, Wang N (2008). HDL, ABC transporters, and cholesterol efflux: implications for the treatment of atherosclerosis.. Cell Metab.

[7] Zannis VI, Chroni A, Krieger M (2006). Role of apoA-I, ABCA1, LCAT, and SR-BI in the biogenesis of HDL.. J Mol Med.

[8] Calvo D, Gomez-Coronado D, Suarez Y, Lasuncion MA, Vega MA (1998). Human CD36 is a high affinity receptor for the native lipoproteins HDL, LDL, and VLDL.. J Lipid Res.

[9] Connelly MA, Klein SM, Azhar S, Abumrad NA, Williams DL (1999). Comparison of class B scavenger receptors, CD36 and scavenger receptor BI (SR-BI), shows that both receptors mediate high density lipoprotein-cholesteryl ester selective uptake but SR-BI exhibits a unique enhancement of cholesteryl ester uptake.. J Biol Chem.

[10] de Villiers WJ, Cai L, Webb NR, de Beer MC, van der Westhuyzen DR (2001). CD36 does not play a direct role in HDL or LDL metabolism.. J Lipid Res.

[11] Febbraio M, Abumrad NA, Hajjar DP, Sharma K, Cheng W (1999). A null mutation in murine CD36 reveals an important role in fatty acid and lipoprotein metabolism.. J Biol Chem.

[12] Chahil TJ, Ginsberg HN (2006). Diabetic Dyslipidemia.. Endocrinology & Metabolism Clinics of North America.

[13] Weinstock PH, Bisgaier CL, Aalto-Setala K, Radner H, Ramakrishnan R (1995). Severe hypertriglyceridemia, reduced high density lipoprotein, and neonatal death in lipoprotein lipase knockout mice. Mild hypertriglyceridemia with impaired very low density lipoprotein clearance in heterozygotes.. J Clin Invest.

[14] Drover VA, Ajmal M, Nassir F, Davidson NO, Nauli AM (2005). CD36 deficiency impairs intestinal lipid secretion and clearance of chylomicrons from the blood.. J Clin Invest.

[15] Goudriaan JR, den Boer MA, Rensen PC, Febbraio M, Kuipers F (2005). CD36 deficiency in mice impairs lipoprotein lipase-mediated triglyceride clearance.. J Lipid Res.

[16] An P, Freedman BI, Hanis CL, Chen Y-DI, Weder AB (2005). Genome-wide Linkage Scans for Fasting Glucose, Insulin, and Insulin Resistance in the National Heart, Lung, and Blood Institute Family Blood Pressure Program.. Diabetes.

[17] Love-Gregory L, Sherva R, Sun L, Wasson J, Schappe T (2008). Variants in the CD36 gene associate with the metabolic syndrome and high-density lipoprotein cholesterol.. Hum Mol Genet.

[18] Havel RJ, Eder HA, Bragdon JH (1955). The distribution and chemical composition of ultracentrifugally separated lipoproteins in human serum.. J Clin Invest.

[19] Basu SK, Goldstein JL, Anderson GW, Brown MS (1976). Degradation of cationized low density lipoprotein and regulation of cholesterol metabolism in homozygous familial hypercholesterolemia fibroblasts.. Proc Natl Acad Sci U S A.

[20] Feng B, Tabas I (2002). ABCA1-mediated cholesterol efflux is defective in free cholesterol-loaded macrophages. Mechanism involves enhanced ABCA1 degradation in a process requiring full NPC1 activity.. J Biol Chem.

[21] Azhar S, Tsai L, Reaven E (1990). Uptake and utilization of lipoprotein cholesteryl esters by rat granulosa cells.. Biochim Biophys Acta.

[22] Zhang JR, Coleman T, Langmade SJ, Scherrer DE, Lane L (2008). Niemann-Pick C1 protects against atherosclerosis in mice via regulation of macrophage intracellular cholesterol trafficking.. J Clin Invest.

[23] Jones C, Garuti R, Michaely P, Li WP, Maeda N (2007). Disruption of LDL but not VLDL clearance in autosomal recessive hypercholesterolemia.. J Clin Invest.

[24] Chen Z, Fitzgerald RL, Averna MR, Schonfeld G (2000). A targeted apolipoprotein B-38.9-producing mutation causes fatty livers in mice due to the reduced ability of apolipoprotein B-38.9 to transport triglycerides.. J Biol Chem.

[25] Mehrdad H, James BM (1995). 9-cis-retinoic acid increases apolipoprotein AI secretion and mRNA expression in HepG2 cells.. Atherosclerosis.

[26] Park YM, Febbraio M, Silverstein RL (2009). CD36 modulates migration of mouse and human macrophages in response to oxidized LDL and may contribute to macrophage trapping in the arterial intima.. J Clin Invest.

[27] Waddington EI, Boadu E, Francis GA (2005). Cholesterol and phospholipid efflux from cultured cells.. Methods.

[28] Hassan HH, Bailey D, Lee D-YD, Iatan I, Hafiane A (2008). Quantitative Analysis of ABCA1-dependent Compartmentalization and Trafficking of Apolipoprotein A-I.. Journal of Biological Chemistry.

[29] Drover VA, Nguyen DV, Bastie CC, Darlington YF, Abumrad NA (2008). CD36 mediates both cellular uptake of very long chain fatty acids and their intestinal absorption in mice.. J Biol Chem.

[30] Tall AR (2008). Cholesterol efflux pathways and other potential mechanisms involved in the athero-protective effect of high density lipoproteins.. J Intern Med.

[31] Nahle Z, Hsieh M, Pietka T, Coburn CT, Grimaldi PA (2008). CD36-dependent regulation of muscle FoxO1 and PDK4 in the PPAR delta/beta-mediated adaptation to metabolic stress.. J Biol Chem.

[32] Kersten S (2008). Peroxisome proliferator activated receptors and lipoprotein metabolism.. PPAR Res.

[33] Rader DJ (2003). Regulation of reverse cholesterol transport and clinical implications.. Am J Cardiol.

[34] Oram JF, Vaughan AM (2006). ATP-Binding cassette cholesterol transporters and cardiovascular disease.. Circ Res.

[35] Chroni A, Nieland TJ, Kypreos KE, Krieger M, Zannis VI (2005). SR-BI mediates cholesterol efflux via its interactions with lipid-bound ApoE. Structural mutations in SR-BI diminish cholesterol efflux.. Biochemistry.

[36] Srivastava RA (2000). Apolipoprotein E gene expression is reduced in apolipoprotein A-I transgenic mice.. Mol Cell Biochem.

[37] Malik S (2003). Transcriptional regulation of the apolipoprotein AI gene.. Front Biosci.

[38] Wada T, Gao J, Xie W (2009). PXR and CAR in energy metabolism.. Trends Endocrinol Metab.

[39] Johnson LA, Altenburg MK, Walzem RL, Scanga LT, Maeda N (2008). Absence of hyperlipidemia in LDL receptor-deficient mice having apolipoprotein B100 without the putative receptor-binding sequences.. Arterioscler Thromb Vasc Biol.

[40] Linder MD, Mayranpaa MI, Peranen J, Pietila TE, Pietiainen VM (2009). Rab8 regulates ABCA1 cell surface expression and facilitates cholesterol efflux in primary human macrophages.. Arterioscler Thromb Vasc Biol.

[41] Sun B, Boyanovsky BB, Connelly MA, Shridas P, van der Westhuyzen DR (2007). Distinct mechanisms for OxLDL uptake and cellular trafficking by class B scavenger receptors CD36 and SR-BI.. J Lipid Res.

[42] Gu X, Kozarsky K, Krieger M (2000). Scavenger receptor class B, type I-mediated [3H]cholesterol efflux to high and low density lipoproteins is dependent on lipoprotein binding to the receptor.. J Biol Chem.

[43] Gu X, Trigatti B, Xu S, Acton S, Babitt J (1998). The efficient cellular uptake of high density lipoprotein lipids via scavenger receptor class B type I requires not only receptor-mediated surface binding but also receptor-specific lipid transfer mediated by its extracellular domain.. J Biol Chem.

[44] Connelly MA, Williams DL (2004). Scavenger receptor BI: a scavenger receptor with a mission to transport high density lipoprotein lipids.. Curr Opin Lipidol.

[45] Ji Y, Wang N, Ramakrishnan R, Sehayek E, Huszar D (1999). Hepatic scavenger receptor BI promotes rapid clearance of high density lipoprotein free cholesterol and its transport into bile.. J Biol Chem.

[46] Ueda Y, Royer L, Gong E, Zhang J, Cooper PN (1999). Lower plasma levels and accelerated clearance of high density lipoprotein (HDL) and non-HDL cholesterol in scavenger receptor class B type I transgenic mice.. J Biol Chem.

[47] Arai T, Rinninger F, Varban L, Fairchild-Huntress V, Liang CP (1999). Decreased selective uptake of high density lipoprotein cholesteryl esters in apolipoprotein E knock-out mice.. Proc Natl Acad Sci U S A.

[48] Zhou J, Febbraio M, Wada T, Zhai Y, Kuruba R (2008). Hepatic fatty acid transporter Cd36 is a common target of LXR, PXR, and PPARgamma in promoting steatosis.. Gastroenterology.

[49] Sviridov D (2009). Maturation of apolipoprotein A-I: unrecognized health benefit or a forgotten rudiment?. J Lipid Res.

[50] Neufeld EB, Stonik JA, Demosky SJ, Knapper CL, Combs CA (2004). The ABCA1 transporter modulates late endocytic trafficking: insights from the correction of the genetic defect in Tangier disease.. J Biol Chem.

[51] Liu P, Bartz R, Zehmer JK, Ying Y, Anderson RG (2008). Rab-regulated membrane traffic between adiposomes and multiple endomembrane systems.. Methods Enzymol.

[52] Zehmer JK, Huang Y, Peng G, Pu J, Anderson RG (2009). A role for lipid droplets in inter-membrane lipid traffic.. Proteomics.

[53] Ducharme NA, Bickel PE (2008). Lipid droplets in lipogenesis and lipolysis.. Endocrinology.

[54] Brasaemle DL, Dolios G, Shapiro L, Wang R (2004). Proteomic analysis of proteins associated with lipid droplets of basal and lipolytically stimulated 3T3-L1 adipocytes.. J Biol Chem.

[55] Collot-Teixeira S, Barbatis C, Bultelle F, Koutouzis M, Pasterkamp G (2008). CD36 is significantly correlated with adipophilin in human carotid lesions and inversely correlated with plasma ApoAI.. J Biomed Biotechnol.

[56] Buers I, Robenek H, Lorkowski S, Nitschke Y, Severs NJ (2009). TIP47, a Lipid Cargo Protein Involved in Macrophage Triglyceride Metabolism.. Arterioscler Thromb Vasc Biol.

[57] Larigauderie G, Furman C, Jaye M, Lasselin C, Copin C (2004). Adipophilin enhances lipid accumulation and prevents lipid efflux from THP-1 macrophages: potential role in atherogenesis.. Arterioscler Thromb Vasc Biol.

