# Supplementary material for: Enhanced Hepatic apoA-I Secretion and Peripheral Efflux of Cholesterol and Phospholipid in CD36 Null Mice
Source: PLoS One. 2010 Mar 26;5(3):e9906. doi: 10.1371/journal.pone.0009906 (PMC2845618; doi:10.1371/journal.pone.0009906)
Supplement: Table S1 — (0.04 MB DOC) [file pone.0009906.s001.doc]

**Supporting Information**

**Table S1. Real-time PCR primers sequences and references.**

| **Sequence Name** | **Sequence (5'-3')** | **References** |  |
| --- | --- | --- | --- |
| mCD36_F | GGA ACT GTG GGC TCA TTG C | [1] |  |
| mCD36_R | CAT GAG AAT GCC TCC AAA CAC |  |
| mLXRα_F | GCC TCA ATG CCT GAT GTT TC | [1] |  |
| mLXRα_R | CTG CAT CTT GAG GTT CTG TCT TC |  |
| mPXR_F | CAA GGC CAA TGG CTA CCA | [1] |  |
| mPXR_R | CGG GTG ATC TCG CAG GTT |  |
| mABCA1_F | CCT CAG CCA TGA CCT GCC TTG TAG | [2] |  |
| mABCA1_R | CCG AGG AAG ACG TGG ACA CCT TC |  |
| mABCG1_F | CAA CGT GGA TGA GGT TGA GA | [3] |  |
| mABCG1_R | CTG GGC CTC TGT GAA GTT GT |  |
| mApoA-I_F | CTC CTC CTT GGG CCA ACA | [4] |  |
| mApoA-I_R | TGA CTA ACG GTT GAA CCC AGA GT |  |
| mApoA-IV_F | ATG CCA AGG AGG CTG TAG AA |  |  |
| mApoA-IV_R | CAG TTT CCT GGG CTA GAT GC |  |
| mHNF4_F | CCT GAT GCA AGA ACA CAT GG |  |  |
| mHNF4_R | TGG CAG GAG CTT GTA GGA TT |  |  |
| mHMGCR_F | CTT GTG GAA TGC CTT GTG ATT G | [3] |  |
| mHMGCR_R | AGCCGAAGCAGCACAATGT |  |
| mCPT1b_F | TTTGGGAACCACATCCGCCAA |  |  |
| mCPT1b_R | TTATGCCTGTGAGCTGGCCAC | [5] |  |
|  |  |  |  |
| **References**  1. Zhou J, Febbraio M, Wada T, Zhai Y, Kuruba R, et al. (2008) Hepatic fatty acid transporter Cd36 is a common target of LXR, PXR, and PPARgamma in promoting steatosis. Gastroenterology 134: 556-567.  2. Feng B, Tabas I (2002) ABCA1-mediated cholesterol efflux is defective in free cholesterol-loaded macrophages. Mechanism involves enhanced ABCA1 degradation in a process requiring full NPC1 activity. J Biol Chem 277: 43271-43280.  3. Burgess BL, Parkinson PF, Racke MM, Hirsch-Reinshagen V, Fan J, et al. (2008) ABCG1 influences the brain cholesterol biosynthetic pathway but does not affect amyloid precursor protein or apolipoprotein E metabolism in vivo. J Lipid Res 49: 1254-1267.  4. Huuskonen J, Vishnu M, Chau P, Fielding PE, Fielding CJ (2006) Liver X receptor inhibits the synthesis and secretion of apolipoprotein A1 by human liver-derived cells. Biochemistry 45: 15068-15074.  5. Gentile L, Monti M, Sebastiano V, Merico V, Nicolai R, et al. (2004) Single-cell quantitative RT-PCR analysis of Cpt1b and Cpt2 gene expression in mouse antral oocytes and in preimplantation embryos. Cytogenet Genome Res 105: 215-221. | | |  |
