# Supplementary material for: Enhanced Hepatic apoA-I Secretion and Peripheral Efflux of Cholesterol and Phospholipid in CD36 Null Mice
Source: PLoS One. 2010 Mar 26;5(3):e9906. doi: 10.1371/journal.pone.0009906 (PMC2845618; doi:10.1371/journal.pone.0009906)
Supplement: Figure S1 — (0.05 MB DOC) [file pone.0009906.s002.doc]

**Figure S1**. Cell membrane ABCA1 distribution in primary hepatocytes isolated from WT and CD36^-/-^ mice. The biotinylation experiment was conducted as described in the Methods section. Membrane Cadherin 1 (CDH 1) was used as a positive control. The ratio of relative signal intensity between ABCA1 and CDH 1 within each sample is listed at the bottom, N=3.

**WT CD36^-/-^**


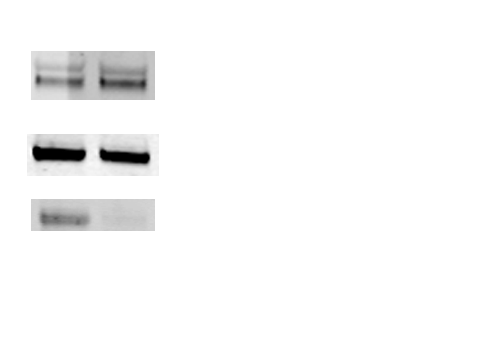


**ABCA1**

**CDH 1**

**CD36**

**0.22 ± 0.12 0.49 ± 0.17 ABCA1/CDH 1 Ratio**
