# Supplementary material for: Enhanced Hepatic apoA-I Secretion and Peripheral Efflux of Cholesterol and Phospholipid in CD36 Null Mice
Source: PLoS One. 2010 Mar 26;5(3):e9906. doi: 10.1371/journal.pone.0009906 (PMC2845618; doi:10.1371/journal.pone.0009906)
Supplement: Figure S2 — (0.03 MB DOC) [file pone.0009906.s003.doc]

**Figure S2. Expression of cell type-specific mRNA in cells isolated from WT and CD36^-/-^ mice**. To examine whether primary hepatocyte preparations contained a significant number of contaminating macrophages, we quantified expression by RT-PCR (see methods section) of the macrophage (and kupfer cells) membrane marker F4/80. The results show that isolated primary hepatocytes have very low to undetectable levels of contaminating macrophages and kupfer cells. Data shown are from WT mice and are similar to those obtained from CD36^-/-^ mice.
