# Supplementary material for: Enhanced Hepatic apoA-I Secretion and Peripheral Efflux of Cholesterol and Phospholipid in CD36 Null Mice
Source: PLoS One. 2010 Mar 26;5(3):e9906. doi: 10.1371/journal.pone.0009906 (PMC2845618; doi:10.1371/journal.pone.0009906)
Supplement: Figure S3 — (0.10 MB DOC) [file pone.0009906.s004.doc]

**Figure S3. Levels of the lipid droplet associated proteins TIP47 and ADRP in primary macrophages from WT and CD36^-/-^ mice**. Proteins in macrophage extracts pooled from 4 mice per genotype were separated by SDS-PAGE, blotted and probed for TIP47 and ADRP (antibodies were a kind gift of Dr. N. Wolins). Detection was by infrared secondary antibodies. The western shows a significant decrease in protein levels of ADRP (but not TIP47) in macrophages from CD36^-/-^ mice.

**TIP47**

**ADRP**

**β-Actin**


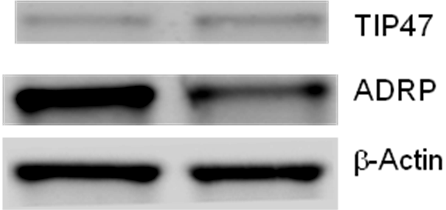

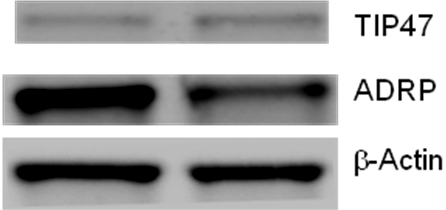


**WT CD36^-/-^**
